# Measurement of Serum *EGFR mRNA* Expression is a Reliable Predictor of Treatment Response and Survival Outcomes in Non- Small Cell Lung Cancer

**DOI:** 10.31557/APJCP.2020.21.11.3153

**Published:** 2020-11

**Authors:** Anant Mohan, Ashraf Ansari, Mirza Masroor, Alpana Saxena, R M Pandey, Ashish Upadhyay, Kalpana Luthra, G C Khilnani, Deepali Jain, Rakesh Kumar, Randeep Guleria

**Affiliations:** 1 *Department of Pulmonary Medicine and Sleep Disorders, All India Institute of Medical Sciences, New Delhi, India. *; 2 *Department of Biochemistry, Maulana Azad Medical College, New Delhi, India. *; 3 *Department of Biostatistics, All India Institute of Medical Sciences, New Delhi. *; 4 *Department of Pathology, All India Institute of Medical Sciences, New Delhi, India. *; 5 *Department of Nuclear Medicine, All India Institute of Medical Sciences, New Delhi, India. *

**Keywords:** EGFR expression, lung cancer, survival

## Abstract

**Background:**

*EGFR* over-expression plays a key role in the development and progression of lung cancer. However, its status as a prognostic biomarker for survival outcomes is unclear.

**Objectives:**

To evaluate the prognostic utility of serum *EGFR mRNA* expression in Non-Small cell lung cancer (NSCLC) for treatment response and survival.

**Methods:**

*EGFR mRNA* levels were determined in serum using quantitative reverse transcriptase polymerase chain reaction (qRT-PCR). Based on ROC curve, a cut off value of 16.0-fold increase was selected to categorize patients into low EGFR (≤ 16.0) and high EGFR (> 16.0) groups.

**Results:**

A total of 350 subjects were included (78.3% males), with mean (± SD) age of 57.1 (± 11.2) years, and including 247 (70.6%) adenocarcinoma (ADC). Majority (73.1%) had metastatic (stage IV) disease. Patients had higher pre-treatment serum EGFR mRNA levels than controls [median fold-increase (min, max), 16.2 (1.9, 66.7). Serum EGFR mRNA levels significantly reduced in those who achieved objective response and disease control. Significantly longer OS and PFS was observed in subjects having baseline *EGFR mRNA* expression ≤ 16.0 fold- increase compared to those with > 16.0 fold- increase [median (95% CI) OS: 25.0 (14.9, NR) versus 7.7 (6.3, 8.9) months; HR (95% CI) 2.9 (2.3, 4.0), p< 0.001; and PFS: 9.9 (7.1, 11.5) versus 6.0 (4.1, 7.5) months; HR (95% CI) 1.8 (1.3, 2.4), p< 0.001].

**Conclusion:**

Serum *EGFR mRNA* expression is a useful parameter for predicting treatment response and survival outcomes in NSCLC.

## Introduction

The Epidermal Growth Factor Receptor (EGFR) appears to play a key role in the development and progression of various cancers, including lung. Alterations in *EGFR* in the form of overexpression, amplification, or mutations are postulated to contribute to the development or propagation of lung cancer by enhancing cellular proliferation, apoptosis inhibition, and angiogenesis (Ciardiello and Tortora, 2003; Gazdar, 2009). However, existing literature regarding prognostic role of *EGFR *expression remains inconsistent, with some reports indicating that *EGFR *expression is associated with poor survival (Li et al., 2008; Sun et al., 2015; Chang et al., 2017) while others have reported no prognostic association (Hirsch et al., 2017; Brabender et al., 2001). Furthermore, most existing studies have been done in the resected tissue specimens of early stage lung cancer (Nakamura et al., 2006; Meert et al., 2002). However, in most countries including India, lung cancer is diagnosed at a relatively advanced stage when they are not candidates for surgical resection, hence resected specimens are unavailable (Chandra et al., 2009; Guleria et al., 1997; Jindal, 1990). In this regard, blood (serum / plasma) is being considered an attractive alternative to tissue for *EGFR* expression testing (Lee, 2006; Zhuo et al., 2014). Since sparse literature exists regarding the value of *EGFR* expression in blood of advanced stage lung cancer patients, the prognostic significance of *EGFR* expression in lung cancer and, more importantly, its ability to predict response to anti-EGFR therapies, are currently subjects of active research. In an attempt to evaluate the above-mentioned lacunae, the present study was planned with an aim to (a) evaluate the prognostic utility of serum *EGFR mRNA* expression in Non-Small cell lung cancer (NSCLC).

## Materials and Methods

*Patients and Methods*

This was a prospective observational study conducted from June 2015 till 31^st^ December 2017 at a tertiary level academic health centre in North India. Newly diagnosed, treatment naïve patients with Non-small cell lung cancer (NSCLC) were included. Patients with concurrent cancer at any other site, or those who were planned for surgery or who had previously received any form of chemotherapy were excluded. The control group included 50 healthy subjects matched for age, sex, and smoking status and without any clinical evidence of malignant disease and no recent acute infection (within the past six weeks).

After obtaining informed written consent, relevant clinical and demographic details were entered a pre-designed structured proforma. For smoking burden, one pack-year was calculated by considering one pack having 20 cigarettes smoked every day for 1 year. For bidi smokers (a local form of tobacco inhalation through rolled tendu leaves), smoking index (S.I.) was calculated by multiplying the number of bidis smoked per day multiplied by number of years smoked (Shimkhada and Peabody, 2003; Singh et al., 2012; Jindal et al., 1982; Reddy and Reddy, 2004) Ex / reformed smokers were defined as those who had quit smoking at least six months previously. Performance status was assessed using a) Karnofsky Performance Scale (KPS), (Karnofsky and JH, 1949) and b) Eastern Co-operative Oncology Group (ECOG) Scale (Oken et al., 1982). In addition, history of prior anti-tubercular treatment was enquired, especially if taken during the current episode of symptoms. Details of diagnostics and staging investigations were recorded. Staging was done according to American Joint Committee for Cancer Control (AJCC) -TNM staging scheme (7^th ^edition) upto 31^st^ December 2016 and as per the joint American Joint Committee for Cancer Control (AJCC) – International Association for Study of Lung Cancer (IASLC) – Union for International Cancer Control (UICC) - 8th edition recommendations from 1^st^ January 2017 (Brierley et al., 2017; Amin et al., 2017; Rami-Porta, 2016; Detterbeck et al., 2017). 

Peripheral venous blood sample (3 ml) was collected in a plain vial from each patient before initiating treatment, and from the control subjects, after written informed consent. The sample was left standing for 20-30 minutes at room temperature and allowed to clot. Serum was separated by centrifugation of blood sample at 1,800×g for 10 min. and removed by Pasteur pipette in a separate eppendorf tube. The serum was stored at -80°C until further analysis. 

*Determination of EGFR mRNA Expression in serum*

Quantitative real -time polymerase chain reaction (qRT-PCR) was carried out to quantify *EGFR* expression by measuring the levels of EGFR mRNA in serum of NSCLC patients and healthy controls, using specific set of primers and SYBR green I technology in the Rotor-Gene Q Real-time PCR cycler (QIAGEN). *EGFR mRNA *expression was determined using the relative quantification PCR double delta cycle threshold (ΔΔCt) method (Livak and Schmittgen, 2001)as per the following steps: (1) RNA extraction using Trizol method; (Chomczynski and Mackey, 1995)(2) cDNA synthesis using cDNA synthesis kit (Thermo Scientific, USA); and (3) verification of cDNA integrity using β actin gene as internal control. The PCR products were analysed on 2.0% agarose gel, stained with ethidium bromide, visualized and photographed in a ultraviolet (UV) Gel documentation system. Quantitative Real Time PCR (qPCR) for EGFR and β-Actin was performed using specific primers (Bieche et al., 2003). 

*Calculation of EGFR Expression*

The Ct or threshold cycle value is the number of cycles required for the fluorescent signal to cross the threshold. Ct levels are inversely proportional to the amount of target nucleic acid in the sample. The expression level is calculated as per the following formula:

1) Normalize Ct* (Target gene) to Ct (reference gene)

ΔCt (Control) = Ct (target gene) - Ct (reference gene)

ΔCt (Patient) = Ct (target gene) - Ct (reference gene)

2) Normalize ΔCt of patient sample to ΔCt of healthy control.

ΔΔCt = ΔCt (Patient) - ΔCt (control)

3) Calculate expression ratio or fold difference.

Ratio/Fold change = 2^-ΔΔCt^

(*Ct = Cycle threshold)

Serum* EGFR* expression was determined at 3 months following treatment either with oral Gefitinib tablet (250 mg once daily in the subgroup of tissue EGFR mutation-positive patients), or with conventional chemotherapy (four cycles of injectable Carboplatin plus Paclitaxel at 3-weekly intervals in patients with tissue-negative EGFR mutations and Squamous cell cancer). Clinical response was evaluated after 2-3 months in Gefitinib group and after 4 cycles of chemotherapy in the chemotherapy group by repeat imaging with CT scan of chest and upper abdomen or by 18F- Fluorodeoxyglucose (FDG) PET/CT scanning. Treatment response was assessed using the RECIST 1.1 criteria (Eisenhauer et al., 2009)and classified as one of the following: Complete Response (CR); Partial response (PR); Progressive Disease (PD); or Stable Disease (SD). Subsequently, Objective Response Rate (ORR) was calculated as the proportion of subjects with a tumor size reduction of a predefined for a minimum time period defined as the sum of Complete Response (CR) plus Partial response (PR) (Pazdur, 2008). Disease Control Rate (DCR) was defined as the sum of Complete Response (CR), Partial response (PR), and Stable disease (SD) (Nakashima et al., 2016; Claret et al., 2014). For the purpose of analysis, subjects who achieved CR or PR were termed as “Responders”, while those with SD or PD were termed “Non-responders”.

*Evaluation of survival parameters*

All patients were followed up using appropriate imaging (CT-PET scans / CT chest) every 3-4 months for a period of one year from date of diagnosis. Subjects were asked to follow up periodically in the clinic; those who missed their visits were contacted by telephone to ascertain their clinical status and all relevant clinical details were recorded; in case of death of the patient, the date of death was recorded. In situations where no contact could be established after repeated phone calls, the last date of known contact by phone or by hospital visit was used for calculating survival outcome parameters. Last date of follow-up was kept as 31^st^ December 2017 and data was censored at this date. The following clinical outcomes were determined: (a) Overall survival (OS): Defined as the time period from date of diagnosis till date of death or last date of known follow – up (US Dept. of Health, 2007). The patients who survived at the time of study closure had their survival time censored to that date; and (b) Progression free survival (PFS): Defined as the time from date of starting treatment till the date of documented disease progression or death, or last date of follow up. The patients who were progression - free at the time of study closure had their time to progression censored to that date.

The sample size of the study was computed to determine the role of serum *EGFR mRNA* expression at baseline on improvement in survival in patients on treatment, at 12 months based on the initial data obtained at 12 months follow up of the first 50 patients in the study. Keeping an estimated proportion of 1-year survival in the low* EGFR* expression group as 75% and 50% in the high *EGFR* expression group, with hazard ratio (HR) of 1.94, 80% power and alpha – error of 5%, the required number of subjects in each of the 2 groups (low / high EGFR) were estimated to be 61. Considering that approx. 1/3 of the patients would not be initiated on treatment and some patients may be censored during follow-up, we would have to enrol minimum 250 subjects in total. 50 normal age, gender and smoking matched control subjects were included for comparison of serum *EGFR* expression.

**Table 1:** Demographic Characteristics of the Study Subjects

Variable	Adenocarcinoma (n=247)	Squamous cell (n=103)	Total (n=350)
Age (years)	55.4 ± 11.6	60.9 ± 9.0	57.1 ± 11.2
Duration of symptoms (days)	155.2 ± 109.1	173.1 ± 125.9	160.4 ± 114.4
	120 (75.40)	150 (75.40)	120 (75.40)
Age category			
≤ 50 years	89 (36.1)	14 (13.6)	103 (29.4)
51 – 65 years	114 (46.1)	59 (57.3)	173 (49.4)
≥ 66 years	44 (17.8)	30 (29.1)	74 (21.2)
Gender	178 (72.1)	96 (93.2)	
Males	69 (27.9)	7 (6.8)	274 (78.3)
Females			76 (21.7)
Smoking Status			
Non-smokers	105 (42.1)	7 (6.8)	112 (31.7)
Current / ex- smokers	142 (57.9)	96 (93.2)	238 (68.3)
Smoking Index (n=238) *			
≤ 250	50 (35.3)	22 (22.9)	72 (30.3)
251 – 500	44 (30.9)	27 (28.1)	71 (29.8)
>500	48 (33.8)	47 (49.0)	95 (39.9)
Stage			
Non-metastatic (I-III)	48 (19.4)	46 (44.7)	94 (26.9)
Metastatic (IV)	199 (80.6)	57 (55.3)	256 (73.1)
KPS			
40 – 70	100 (40.5)	53 (51.5)	153 (43.7)
80 – 100	147 (59.5)	50 (48.5)	197 (56.3)
ECOG score			
0 / 1	138 (55.9)	52 (50.5)	190 (54.3)
2	82 (33.2)	38 (36.9)	120 (34.3)
3	27 (10.9)	13 (12.6)	40 (11.4)

All values expressed as number (%) , median (min, max), or mean ± SD; * Excluding lifetime non-smokers. Smoking index was calculated by multiplying the number of bidis smoked per day with the number of years smoked

**Figure 1:**
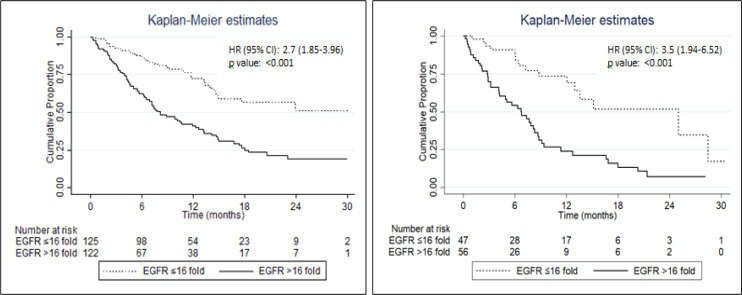
Overall Survival Time in Adenocarcinoma (panel A) and Squamous Cell (Panel B) Lung Cancer Subjects Having low (≤ 16.0) and high (> 16.0) Baseline Serum EGFR mRNA Levels. OS, overall survival; HR, hazard ratio; CI, confidence interval; NR, not reached; EGFR values expressed as fold-increase over control values

**Table 2:** Serum EGFR mRNA Expression at Baseline and after Treatment

Histology type	Parameter	Baseline levels (fold - increase over controls)	Post treatment levels	p-value	Absolute difference (pre-post)	% difference
Adeno	Mean ± SD	17.7 ± 8.2	11.5 ± 8.2	<0.001	4.3 ± 10.8	9.9 ± 76.9
	Median	16	9.2		4.2	34.3
	(min, max)	(1.9, 66.7)	(3.4, 56.5)		(-40.3, 62.2)	(-340.7, 93.2)
	n	247	118		118	
Squamous	Mean ± SD	17.5 ± 7.7	11.5 ± 8.0	<0.001	5.1 ± 9.2	20.9 ± 59.2
	Median	16.9	9.3		5.6	36.7
	(min, max)	(4.1, 42.8)	(2.5, 41.8)		(-16.7, 33.6)	(-178.9, 78.4)
	n	103	43		43	43
p value		0.94	0.81		0.72	0.39
Total (all subjects)	Mean ± SD	17.6 ± 8. 1	11.5 ± 8.1	<0.001	4.5 ± 10.4	12.8 ± 72.6
	Median	16.2	9.2		4.45	34.9
	(min, max)	(1.9, 66.7)	(2.5, 56.5)		(-40.3, 62.2)	(-340.8, 93.3)
	n	350	161		161	161

All values expressed as mean ± SD, or median (min, max)

**Figure 2:**
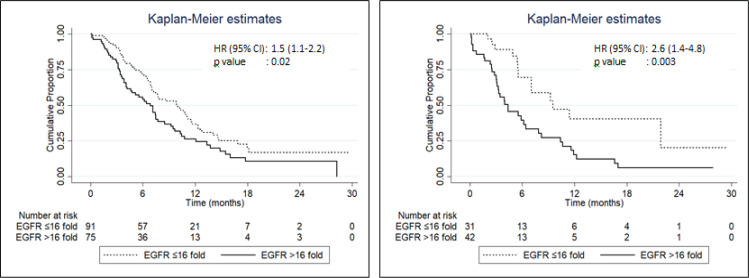
Progression-Free Survival Time in Adenocarcinoma (Panel A) and Squamous Cell (Panel B) Lung Cancer Subjects Having Low (≤ 16.0) and High (> 16.0) Baseline Serum EGFR mRNA Levels. PFS, Progression-free survival; HR, hazard ratio; CI, confidence interval; NR, not reached; EGFR values expressed as fold-increase over control values

**Table 3:** Change in Serum EGFR mRNA Expression Levels Based on Treatment Modality Received

Treatment modality		Baseline (Pre- treatment) EGFR mRNA expression (fold- increase)	Post treatment EGFR mRNA expression (fold- increase)	p value	Absolute reduction (pre – post)
No treatment	N	83	n=0	----	n=0
	Mean ± SD	19.6 ± 9.5			
	Median	17.5			
	(min, max)	(4.1, 56.1)			
Chemotherapy	N	203	120	<0.001	120
	Mean ± SD	16.9 ± 7.1	11.4 ± 7.8		4.3 ± 8.9
	Median	15.7	9.2		4.7
	(min, max)	(2.3, 42.8)	(2.4, 56.5		(-37.2, 33.6)
TKI	N	64	41	0.001	41
	Mean ± SD	17.1 ± 8.6	11.8 ± 9.2		4.9 ± 13.9
	Median	16.6	9.2		4.2
	(min, max)	(1.9, 66.7)	(3.8, 56.5)		(-40.3, 62.2)
	p-value	0.03	0.94		0.74

All values expressed as mean ± SD, or median (min, max)

**Figure 3:**
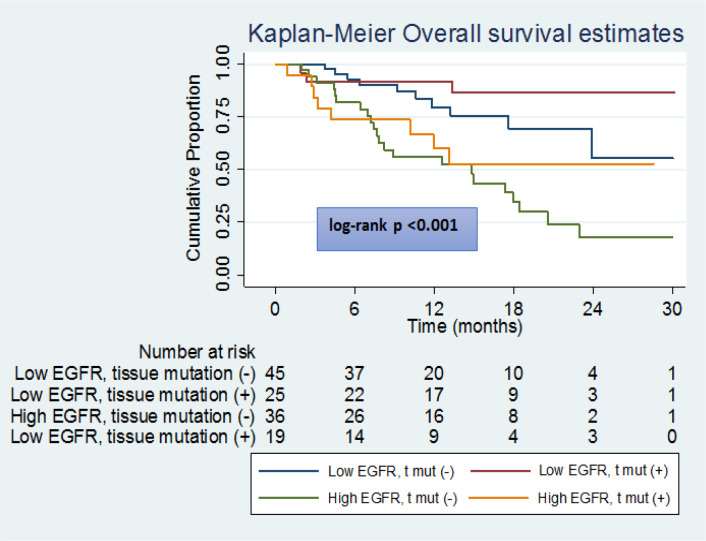
OS in Patients with High and Low Baseline EGFR mRNA Levels Based on Their Tissue Mutation Status

**Table 4:** Serum EGFR mRNA Levels based on Objective Response, Disease Control and Mortality in NSCLC

Variable		Pre- treatment serum EGFR mRNA levels (fold-increase)	Post -treatment serum EGFR mRNA levels	p-value
Treatment Response	N	102	97	<0.001
Responders	Mean ± SD	15.7 ± 8.1	10.2 ± 6.9	
	Median	14.9	8.6	
	(min, max)	(1.9, 66.7)	(2.5, 56.5)	
Non-responders				
	N	72	64	0.009
	Mean ± SD	16.5 ± 7.3	13.5 ± 9.4	
	Median	15.3	10.9	
	(min, max)	(2.3, 38.5)	(2.5, 56.5)	
	p-value	0.39	0.01	
Disease Control	N	126	120	<0.001
Yes	Mean ± SD	15.8 ± 7.7	9.8 ± 6.3	
	Median	14.8	8.7	
	(min, max)	(1.9, 66.7)	(2.5, 56.5)	
No	p-value	48	41	0.62
	Mean ± SD	16.8 ± 8.0	16.2 ± 10.7	
	Median	15.8	12.3	
	(min, max)	(1.9, 66.7)	(2.9, 56.5)	
	p-value	0.48	<0.001	
Mortality	N	179	48	<0.001
Yes	Mean ± SD	21.1 ± 8.5	11.8 ± 9.6	
	Median	19.3	8.9	
	(min, max)	(8.3, 66.7)	(4.1, 56.5)	
No	N	171	113	<0.001
	Mean ± SD	13.9 ± 5.7	11.4 ± 7.5	
	Median	13.6	9.7	
	(min, max)	(1.9, 27.1)	(2.5, 56.5)	
	p-value	<0.001	0.62	

All values expressed as mean ± SD or median (min, max)

**Figure 4:**
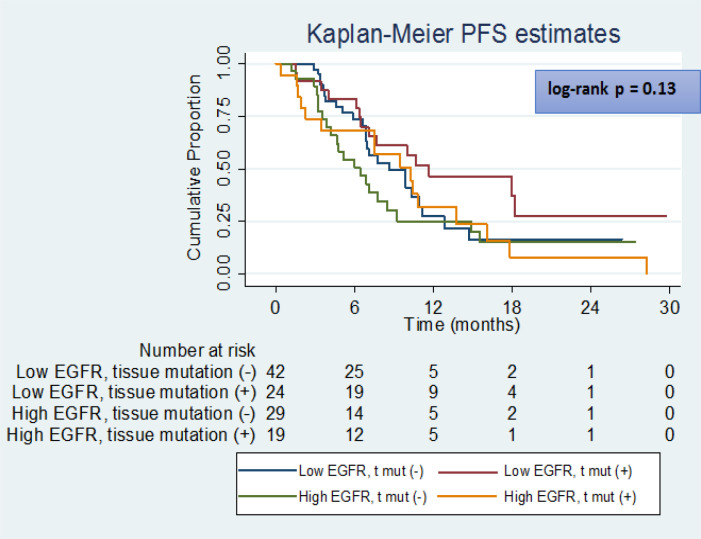
PFS of Patients with High and Low Baseline EGFR mRNA Levels based on Their Tissue Mutation Status

## Results

During the study period, a total of 395 subjects were screened, of whom 45 were subsequently excluded (previous / ongoing chemotherapy =18; refusal of consent=16; tumor morphology not defined=11). Finally, 350 subjects were included for evaluation and analysis. Of these, 247 (70.6%) had adenocarcinoma (ADC) and 103 (29.4%) had squamous cell carcinoma (SCC) morphology. The demographic characteristics of the study group is shown in Table 1. The study group comprised 274 males (78.3%) overall, with mean (SD) age of 57.1 (11.2) years. Majority of subjects (68.3%) were either current or reformed smokers. The mean (SD) symptom duration of the study group was 160.4 (114.4) days, with median duration of 120 days (range, 7 to 540 days). 

Among the total 350 subjects, 256 (73.1%) had metastatic (stage IV) disease (80.6% of ADC and 55.3% of SCC). The commonest metastatic sites were the lungs (28.9%), pleural effusion (26.9%), and bone (26%), followed by brain (14%), adrenals (12.3%), and liver (7.4%). Other metastatic sites included extra thoracic lymph nodes (n=12), and kidney, parotid, pancreas and thyroid (1 patient each).

Of the total 350 subjects, 267 (76.3%) were initiated on treatment. Of these, 203 (58%) subjects were initiated on chemotherapy (51% in ADC and 74.8% in SCC), while 64 patients (25.9%, all ADC) received oral Tyrosine kinase inhibitors (TKIs) in the form of Gefitinib tablets. The remaining subjects received best supportive care or opted for alternative modalities of treatment. Radiotherapy was administered for palliative purposes to 52 patients (14.9%) and with curative intent in 8 (2.3%) patients. 

Of the total subjects, response to treatment was evaluated in 174 (130 in chemotherapy group and 44 in the Gefitinib group). Overall, majority of patients (57.5%) achieved a partial response (PR) [56.1% in chemotherapy group and 61.3% in the Gefitinib group]. Objective response (OR) was achieved in 102 patients (58.6%) of the total group (57.7% in chemotherapy group and 61.3% in the Gefitinib group) while disease control (DC) was achieved in 126 patients (72.4%) of the total group (71.5% in chemotherapy group and 75.0 % in the Gefitinib group).

By the end of the study follow up period, out of 350 subjects, 179 (51.1%) did not survive. The median overall survival (OS) time was 13.2 months (95% CI, 10.9 to 14.9 months). The OS time of ADC was significantly greater than OS time in SCC [median (95% CI), 14.6 (11.9, 17.6) versus 8.8 (7.3, 13.6) months respectively, log-rank p-value=0.01]. The hazard of mortality in SCC subjects was 1.5 times higher as compared to the ADC subjects [HR (95% CI): 1.5 (1.1, 2.0), p = 0.01].

Disease progression occurred in 163 (68.2%) of 267 patients who were initiated on any treatment. The median PFS time of the whole study group was 7.5 months (95% CI, 6.6-9.2 months). The PFS time of ADC was not significantly different from that of SCC patients [median (95% CI), 7.7 months (6.9, 9.9) vs 6.2 (4.9, 9.5) months respectively); HR (95% CI), 1.2 (0.8, 1.6), p = 0.41]. 

*Serum EGFR mRNA expression of the study group before and after treatment*

*EGFR mRNA *expression was measured in serum at baseline and after treatment in 247 Adenocarcinoma (ADC) and 103 Squamous cell carcinoma (SCC) subjects. Throughout this document, patient EGFR mRNA values are expressed as fold-increase in levels compared to the control values. Figure 1 shows a representative picture of (a) RNA extraction; (b) cDNA synthesis from RNA; (c) RT-PCR amplification graph and (d) and melting curve during EGFR mRNA determination. 

Following treatment, EGFR levels reduced significantly in males as well as females, in all age categories and within each of the three smoker categories (Table 2). Overall, the median (min, max) fold - increase in baseline *EGFR mRNA* expression compared to controls was 16.2 (1.9, 66.7), which reduced significantly following treatment to 9.2 (2.5, 56.5) [p<0.001]. Individually in ADC and SCC, the baseline *EGFR mRNA* expression was elevated and reduced significantly following treatment. However, the EGFR mRNA levels were similar between ADC and SCC groups at baseline as well as following treatment.

No significant difference existed in serum EGFR mRNA levels between ADC and SCC either at baseline [median (min, max), 16.0 (1.9, 66.7) and 16.9 (4.1, 42.8) respectively, p=0.84], or after treatment [median (min, max), 9.2 (3.4, 56.5) and 9.3 (2.5, 41.8) respectively, p=0.76] or between subjects with metastatic disease and non-metastatic disease. Similarly, no association was seen between EGFR mRNA and KPS or ECOG scores. Following treatment, serum EGFR mRNA levels reduced significantly in both the chemotherapy as well as the Gefitinib groups (p<0.001) (Table 3).

*Serum EGFR mRNA expression as a predictor of treatment response*

Pre-treatment serum *EGFR mRNA* levels were similar in responders and non-responders (Table 4). However, post-treatment *EGFR mRNA* was significantly higher in non-responders, i.e., those who did not achieve Objective response (p=0.01). Comparison of pre and post-treatment EGFR mRNA levels revealed that significant reduction occurred in both responders as well as non-responders (p<0.001 and p=0.009 respectively). However, subjects who failed to achieve disease control did not demonstrate reduction in EGFR levels. 

Significant reduction in EGFR mRNA levels was observed from baseline to post-treatment both in the survivors as well as the subjects who did not survive. However, the baseline serum* EGFR mRNA* expression levels in the subjects who died were significantly higher than those who survived (Table 4). 

The Receiver Operating Characteristics (ROC) curve was used to determine predictive value of baseline serum EGFR mRNA levels to predict mortality and a cut off value of 16.0 was selected, as it corresponded to higher sensitivity and specificity. The OS time was significantly greater in subjects with baseline serum *EGFR mRNA* levels ≤ 16.0-fold-increase compared to those with levels > 16.0-fold-increase [median (95% CI) OS, 25.0 (14.9, NR) vs 7.7 (6.3, 8.9) months respectively; HR (95% CI) 2.9 (2.1, 4.0), log-rank p< 0.001]. The patient group with baseline serum *EGFR mRNA* levels > 16.0 had a 2.9 times higher risk of mortality compared to those with baseline EGFR levels ≤ 16.0. Similar results were found in ADC and SCC (Figure 1); ADC and SCC with baseline serum *EGFR* expression > 16.0 had 2.7 times and 3.5 times higher risk of mortality respectively compared to those with baseline expression ≤ 16.0.

Similarly, the PFS time was significantly greater in subjects with baseline serum *EGFR mRNA* expression levels ≤16.0 fold compared to those with levels > 16.0-fold (Figure 2); the subjects with baseline serum *EGFR* expression > 16.0 had a 1.8 times higher risk of disease progression compared to those with expression ≤ 16.0. Similar findings were obtained in subgroups of ADC and SCC (Figure 3A and 3B) with subjects with baseline serum *EGFR mRNA* expression > 16.0 demonstrating a 1.5 and 2.6-times higher risk of disease progression respectively compared to those with expression ≤ 16.0.

## Discussion

Our results show that baseline (pre-treatment) serum EGFR mRNA levels in NSCLC were elevated compared to the control group, and they reduced significantly following treatment. Over-expression of* EGFR* in lung cancer has been documented previously, although most studies used tissue specimens for EGFR testing. In comparison to normal lung tissue and para-cancerous tissue, lung cancer cells show significantly higher *EGFR* expression (Brabender et al., 2001; Li et al., 2011). The degree of *EGFR* expression has been widely variable and ranges between 32%- 81% (Li et al., 2008; Fontanini et al., 1998; Gaber et al., 2014; Rusch et al., 1997). In a meta-analysis of 18 studies, 1,441 of 2,972 (48.5%) patients had positive *EGFR* expression, majority of which were detected using IHC method (Nakamura et al., 2006). 

Among our study patients, serum *EGFR mRNA *expression was similar in ADC and SCC. Most previous studies have shown higher *EGFR* expression in squamous cell morphology compared to adenocarcinoma, (Hirsch et al., 2003; Nakamura et al., 2006; Rusch et al., 1997; Ludovini et al., 2013) although others have shown opposite results (Sun et al., 2015; Han et al., 2015). The proportion of ADC patients in the present study group was much higher, hence this may have contributed to the similar baseline *EGFR mRNA* expression in both histological types.

The patients in the current study did not demonstrate any significant association between serum *EGFR mRNA* expression and various clinical characteristics such as age, gender, smoking status, disease stage or performance status. Results of some previous studies have demonstrated association of *EGFR* expression with patient characteristics such as female sex, (Suwinski et al., 2012; Arfaoui et al., 2014) smoking status, (Zhuo et al., 2014; Arfaoui et al., 2014) and advanced disease stage, (Sun et al., 2015; Arfaoui et al., 2014; Liang et al., 2012; Traynor et al., 2013) while several others have not. (Hirschet al., 2003; Brabender et al., 2001; Han et al., 2015; Liang et al., 2010). In the few studies that evaluated *EGFR* expression in blood, most included early stage disease undergoing surgical resection and did not find significant association between serum *EGFR* expression and clinical characteristics such as age, sex, or pathological subtype (Zhuo et al., 2014; Ciledag et al., 2008; Sasaki et al., 2003; Dziadziuszko et al., 2006). On the other hand, positive correlation of serum EGFR levels (measured by ELISA) with smoking index has been reported (Zhuo et al., 2014).

Following treatment, the patients in the present study showed a significant decline in serum *EGFR mRNA* levels. Subgroup analysis showed that this decline occurred in both males and females, in all age categories, current and ex-smokers, subjects with smoking index > 250, in both adenocarcinoma and squamous cell carcinoma lung cancer, and in subjects with relatively better performance status. The trend of *EGFR* expression following treatment in lung cancer has been sparsely evaluated primarily because most studies were conducted in tumor tissue, hence repeat testing of *EGFR* expression was not feasible. In serum, *EGFR mRNA* levels were found to decline significantly following surgical resection in 56 patients with early stage NSCLC (Zhuo et al., 2014).In our patients, although serum EGFR mRNA declined in patients with metastatic as well as non-metastatic disease, the degree of decline was similar between both groups. This may possibly be due to the fact that even in the non-metastatic group, most patients had stage IIIB disease, hence this group probably did not truly reflect the early stage lung cancer. 

The baseline serum *EGFR mRNA* expression in the Gefitinib group was significantly higher than in the chemotherapy group. Both treatment arms however, showed a significant reduction in *EGFR* expression following treatment; consequently, the post-treatment *EGFR* levels were similar between both groups. The relationship between *EGFR* expression and treatment modality has not been well elucidated. Most previous studies have included surgically resected tissue for* EGFR* expression, and comparisons with any other treatment modality have not been reported. Similarly, data regarding change in *EGFR* expression following treatment is limited to a solitary study that reported significant decline in serum *EGFR* expression following surgical resection in 56 patients (Zhuo et al., 2014). To the best of our knowledge, no existing publication has as yet reported on the change in *EGFR mRNA* expression levels measured in serum following two different treatment modalities in advanced stage NSCLC. Overall, the results of the present study therefore suggest that both chemotherapy and Gefitinib cause a significant reduction in *EGFR* expression in NSCLC. 

We also found that baseline serum *EGFR mRNA* levels did not predict response to treatment or likelihood of achieving disease control (DC). However, subjects who achieved DC demonstrated significant reduction in serum EGFR mRNA levels from baseline, thereby implying a possible prognostic association. On the other hand, reduction in serum EGFR mRNA levels did not differentiate between responders and non-responders. The utility of *EGFR* expression in predicting treatment response and DC has been sparsely reported, and a previous study showed that the probability of treatment response was significantly greater in NSCLC on gefitinib and with high *EGFR* expression compared to those with low expression, although disease control rates were unaffected. In the same study, a subgroup analysis of 14 EGFR mutated patients revealed that higher *EGFR* expression was associated with response to treatment and better PFS compared to non-responders, thereby indicating that serum *EGFR mRNA* expression may be a promising parameter for predicting therapeutic response in NSCLC, especially those who are EGFR mutated. On the other hand, lack of correlation between *EGFR* expression and treatment response or survival was reported in 375 tissue specimens in the Tarceva Lung Cancer Investigation Trial, (Gatzemeier et al., 2007) as well as in a cohort of previously treated Squamous cell lung cancer patients (Chang et al., 2016).

*Serum EGFR mRNA expression as a Predictor of Survival Outcomes*

We found that OS and PFS was significantly higher in all patients having baseline serum *EGFR mRNA* expression ≤ 16.0-fold-increase compared to those with > 16.0-fold-increase. Similar results were obtained by applying this cut-off value to ADC and SCC groups separately. Compared to patients with low baseline serum *EGFR* expression (≤ 16.0), patients of NSCLC with high serum EGFR (> 16.0) had 2.9 times higher risk of mortality and 1.7 times higher risk of disease progression. 

The prognostic role of *EGFR* expression is as yet, uncertain. Majority of studies in fact, have not shown any significant association of *EGFR* expression with survival outcomes (Suwinski et al., 2012; Gatzemeier et al., 2007; Rusch et al., 1993; Murray S et al., 2012; Seyhan et al., 2010). A few reports, albeit of relatively small sample size, have shown that higher* EGFR* expression is associated with better survival (Dziadziuszko et al., 2006; Chang et al., 2016; Shah et al., 2004). In previously treated SCC patients on TKIs, better PFS and OS was seen in patients with high *EGFR* expression (Chang et al., 2016). The explanation for such an association may be that possibly the loss of expression of these receptors is associated with biologic aggressiveness in lung tumors resulting in adverse outcome for patients with such tumors. On the other hand, studies have also reported worse outcomes associated with high *EGFR *expression (Sun et al., 2015; Li et al., 2011; Traynor et al., 2013) reported that high *EGFR* expression had a negative impact on the survival of post- operative NSCLC patients who underwent radiotherapy. They proposed that* EGFR* expression may relate to radiotherapy resistance and impart a poor outcome. Similarly, positive *EGFR* expression in tissue was a significant predictor of reduced 3-year survival in Chinese females (Sun et al., 2015). It is notable, however, that all the above studies were conducted in the tumor tissue of patients with early stage disease, which is an important distinction from the design of the current study wherein *EGFR* expression was measured in the serum of advanced stage patients. 

Among the two meta-analysis that have been conducted to determine the effect of *EGFR* expression on survival outcomes in lung cancer, results have been variable. The first meta-analysis (Meert et al., 2002) included 11 studies and 2185 patients and the result just reached significance (HR 1.13, 95% CI, 1.00–1.28), implying that patients with a tumour not expressing EGFR had a better survival. They reported significant association of *EGFR* expression with survival only with SCC, unlike our results that showed that survival is associated with *EGFR mRNA* expression in both ADC and SCC. 

The second meta-analysis included 18 studies and 2972 patients (Nakamura et al., 2006). Of note, only three of these studies had included patients with stage IV disease while the remaining had included stages I-IIIB. Six of the 18 studies reported shorter survival with *EGFR* over-expression, while two studies showed longer survival. Overall, *EGFR* overexpression was not shown to have significant impact on survival in NSCLC. However, both the above meta-analysis was limited by lack of data for aggregation in few studies, heterogeneity in method of *EGFR* expression testing, and methods of extrapolation of hazard ratios. 

Based on our ROC analysis, the cut-off of baseline serum *EGFR mRNA* expression level gave a predictive value for mortality as 76%. Previously also, some authors have determined cut-off values based on ROC for testing the role of *EGFR* expression in survival prediction (Chang et al., 2017; Sasaki et al., 2003; Dziadziuszko et al., 2006). None of the previous reports, however, specified the area under the ROC based on their derived cut-off values.

This study had some limitations. The patient recruitment was not consecutive and had disproportionately higher adenocarcinoma subjects. However, since the sample size was calculated based on all NSCLC, the recruited number of patients were statistically adequate to achieve the primary outcome. Secondly, of the 267 subjects who were initiated on treatment, response assessment could only be done in 65.2% (174/267) subjects as the remaining were unable to complete the treatment schedule. Thirdly, we did not perform EGFR mRNA measurements in tissue specimens and based our results only on serum levels. Fourth, we had very few patients with early-stage disease; hence a stage-wise comparison of *EGFR* expression between early and late-stage NSCLC was not possible. In spite of these shortcomings, we feel this study provides useful information regarding a promising biomarker that may have prognostic value in patients with advanced NSCLC.

To conclude, serum *EGFR mRNA* expression is increased in NSCLC and declines following treatment; this is a useful parameter for predicting treatment response and survival outcomes. 
